# BRIDGING GAPS IN GERIATRIC EXPERTISE: OREGON ECHO NETWORK’S IMPACT ON INTERPROFESSIONAL EDUCATION AND CARE

**DOI:** 10.1093/geroni/igae098.3814

**Published:** 2024-12-31

**Authors:** Leah Brandis, Anders Herreid-O’Neill, Maryan Carbuccia Abbott, Laura Byerly, Kerri Smith Slingerland

**Affiliations:** OHSU, Portland, Oregon, United States; Oregon Health & Science University, Portland, Oregon, United States; Oregon Health & Science University, Portland, Oregon, United States; Oregon Health & Science University, Portland, Oregon, United States; Oregon Health & Science University, Portland, Oregon, United States

## Abstract

The United States faces a critical shortage of healthcare professionals trained in geriatric care, exacerbated by a rapidly aging population. In response to this pressing need, the Oregon ECHO Network (OEN) has forged partnerships with local and statewide organizations to pioneer a series of ECHO programs aimed at disseminating geriatric care principles across diverse care settings. Over six years, the network’s reach has expanded from primary care to encompass post-acute and long-term care communities, culminating in a portfolio of geriatric care ECHO courses. This presentation will share results from an Older Adult ECHO Impact Report covering 28 cohorts of 11 different older adult-related ECHO programs since 2018. OEN has engaged more than 50 faculty and guest speakers and 881 registrants for older adult health care education alone. Faculty panels are interdisciplinary and have included prescribing clinicians, such as physicians and nurse practitioners, along with occupational therapists, pharmacists, registered nurses, behavioral health professionals, physical therapists, peer support specialists and others. Emergent themes, ongoing gaps, lessons learned and program impacts will be shared from participant surveys and interviews. Highlighted are new collaborations with the Oregon Center of Excellence for Behavioral Health & Aging and the Geriatric Workforce Enhancement Program, underscoring efforts to address gaps in geriatric expertise through innovative educational models.

